# The university münster model surgery system for orthognathic surgery. Part II – KD-MMS

**DOI:** 10.1186/1746-160X-9-2

**Published:** 2013-01-04

**Authors:** Ulrike Ehmer, Ulrich Joos, Thomas Ziebura, Stefanie Flieger, Dirk Wiechmann

**Affiliations:** 1 Department of Orthodontics, University of Münster, Albert-Schweitzer-Campus 1, 48149, Münster, Germany; 2 Department of Maxillofacial Surgery, University of Münster, Albert-Schweitzer-Campus 1, 48149, Münster, Germany; 3 Department of Orthodontics, Medizinische Hochschule Hannover, Carl-Neuberg-Str. 1, 30625, Hannover, Germany

## Abstract

**Background:**

Model surgery is an integral part of the planning procedure in orthognathic surgery. Most concepts comprise cutting the dental cast off its socket. The standardized spacer plates of the KD-MMS provide for a non-destructive, reversible and reproducible means of maxillary and/or mandibular plaster cast separation.

**Methods:**

In the course of development of the system various articulator types were evaluated with regard to their capability to provide a means of realizing the concepts comprised of the KD-MMS. Special attention was dedicated to the ability to perform three-dimensional displacements without cutting of plaster casts. Various utilities were developed to facilitate maxillary displacement in accordance to the planning. Objectives of this development comprised the ability to implement the values established in the course of two-dimensional ceph planning.

**Results:**

The system - KD-MMS comprises a set of hardware components as well as a defined procedure. Essential hardware components are red spacer and blue mounting plates. The blue mounting plates replace the standard yellow SAM mounting elements. The red spacers provide for a defined leeway of 8 mm for three-dimensional movements. The non-destructive approach of the KD-MMS makes it possible to conduct different model surgeries with the same plaster casts as well as to restore the initial, pre-surgical situation at any time. Thereby, surgical protocol generation and gnathologic splint construction are facilitated.

**Conclusions:**

The KD-MMS hardware components in conjunction with the defined procedures are capable of increasing efficiency and accuracy of model surgery and splint construction. In cases where different surgical approaches need to be evaluated in the course of model surgery, a significant reduction of chair time may be achieved.

## Introduction

Model surgery – according to Epker, Stella, Fisch [1]: Definitive Model Surgery, according to Erickson, Bell, Goldsmith [2]: Analytical Model Surgery or with regard to future developments [3-5]: 3D Virtual Treatment Planning – is an integral part of the planning procedure in orthognathic surgery.

The process pursues the following objectives:

Transfer of data gained from lateral ceph analysis (2D) with regard to clinical, photographic and functional findings to a three-dimensional model (3D)

Determination of displacement values for the surgical intervention (yaw, pitch, roll [2])

Gnathologic splint construction

Realization of aspired movements during surgery

Semi-adjustable articulators, mostly SAM-type products, serve for the three-dimensional treatment planning. Three-dimensional alignment of the maxillary dental cast to a reference plane (Axis Orbital Plane in SAM) is achieved through face bow registration [6-10].

In comparison to conventional methods of face bow transfer, three-dimensional virtual computer assisted planning appears to be more accurate [11].

The maxillary dental cast is separated from its socket approximately at the level of the future osteotomy site. Most commonly, the plaster cast is cut with a saw.

According to Bamber et al. [12] the procedure is described as follows: *“After recording the pre-operative position the maxilla was sectioned at the Le Fort I level, just above the apices of the maxillary teeth with an electric band saw and an appropriate amount of plaster removed […] to allow the planned movements of the maxilla.”*

The standardized spacer plates of the KD-MMS provide for a non-destructive, reversible and reproducible means of maxillary and/or mandibular plaster cast separation [13-15].

The displacement values established during model surgery are recorded in written or – in future developments – electronic protocols, which are then made available to the surgeon during the operation.

In maxillary surgery, a transfer of the pre-established values to the patient is usually conducted by applying marks – e. g. small grooves – on the bony surfaces. In addition, mechanical guidance is achieved by using an appropriate gnathologic splint.

A comparison between surgical planning and final result yields a combined error for which an evidence based identification of individual error sources is not yet available. According to Zizelmann et al. [11] “*only the sum of all possible errors (face-bow recording errors, errors in cephalometric technique, changes in position of the condyle, misidentification of the vertical distance, surgical inaccuracies, and so on) is detected.*”

## Methods

The present methodology and results were not based on experimental research carried out on humans or animals. Therefore, an approval of an ethics committee was not necessary.

### Choice of articulator

In the course of development of the system various articulator types were evaluated with regard to their capability to provide a means of realizing the concepts comprised of the KD-MMS.

**Figure 1:**
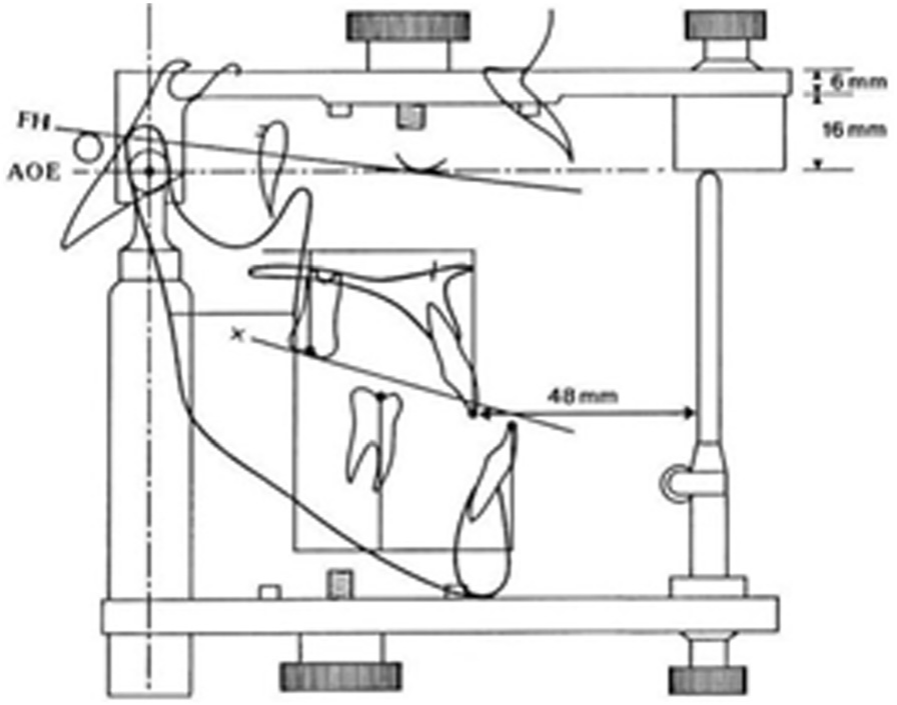
**Graphical overlay of ceph tracing and articulator depicting the common reference plane (Axis Orbital Plane) and the Marker Lines System (AO-MLS) [**[13]**].**

Arcon articulators appear preferrable to Non-Arcon devices in that their construction resembles more closely the actual and radiologically visible anatomical structures (Figure 1).

The SAM 2P (SAM Präzisionstechnik GmbH, Gauting, Germany) is a widely used articulator. It meets the requirements of being Arcon and provides sufficient vertical space.

### System inherent leeway for three-dimensional movements

**Figure 2:**
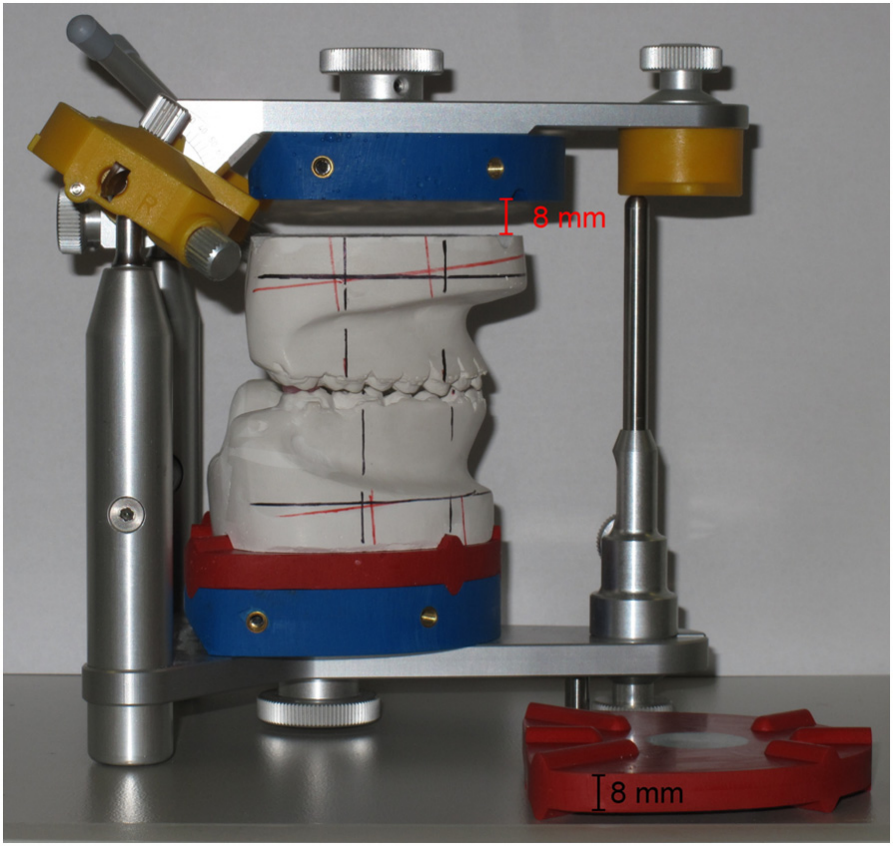
Articulator-mounted plaster models with removed maxillary red spacer plate showing the amount of vertical leeway, note also the retention ridges.

A main objective of the method consists in the ability to perform three-dimensional displacements without cutting of plaster casts. In prior KD-MMS-versions, flat spacer plates of 6 mm thickness were used for this purpose. However, this amount of vertical space proved insufficient in some, especially pronounced long-face cases. In addition to resolving this issue, the 8 mm red spacers used now are equipped with defined triangular retention ridges which permit for a precise repositioning of the plaster casts (Figure 2).

The use of repositionable spacers permits for a non-destructive way of performing model surgery in which all components can be replaced into their initial positions. This provides the possibility to perform multiple model surgeries using one set of plaster casts and improves overall effectiveness.

### Tools for model surgery

**Figure 3:**
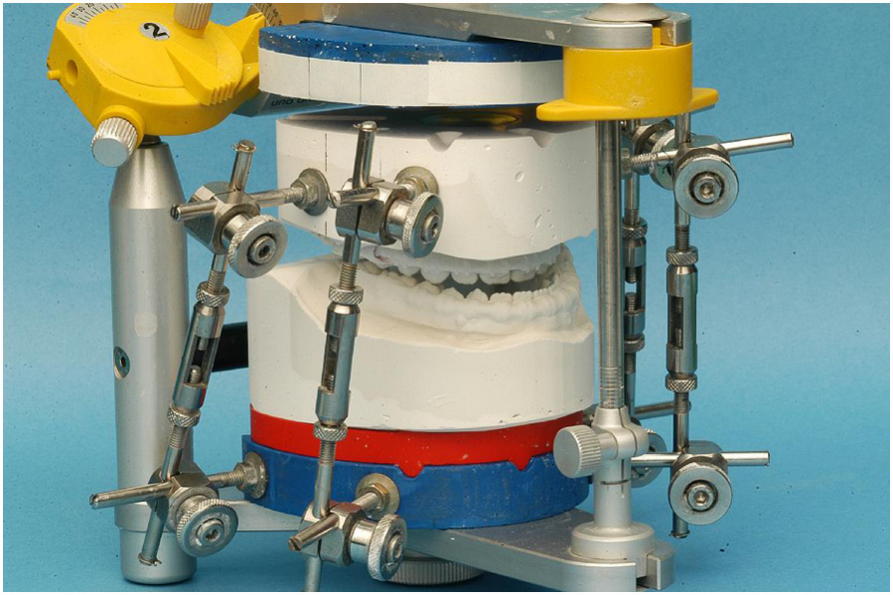
Maxillary model displacement using adjustment rods for three-dimensional positioning.

Various utilities were developed to facilitate maxillary displacement in accordance to the planning [6,16]. Objectives of this development comprised the ability to implement the values established in the course of two-dimensional ceph planning. It should be possible to conduct model surgery without the use of wax and thereby retain a full view of occlusal and basal relations throughout all stages of the process. An example of this concept is shown in Figure 3.

## Results

**Figure 4:**
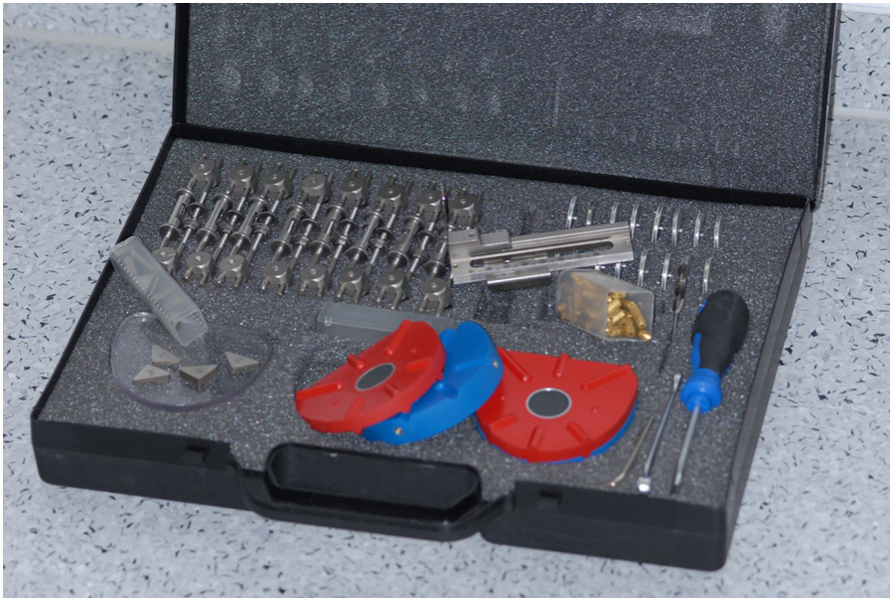
Hardware components of the University Münster Model Surgery System for Orthognathic Surgery (Orbis Will, Ahaus, Germany).

The KD-MMS comprises a set of hardware components (Figure 4) as well as a defined procedure.

### Hardware components

The following compendium provides an overview of the KD-MMS components. Each part of the system is presented with a picture, the component’s name, a short usage description and finally a recommendation on essential or optional application (Table 1).

**Table 1:** KD-MMS components

	**Component**	**Purpose and usage**	**Essential/ optional**
	blue mounting plate	Replaces standard yellow SAM-mounting plate, first part of the double splint system.A SAM-P version is strongly recommended due to its greater vertical leeway.	Essential
	red spacer plate	Completes double splint system.Mounted between blue plate and plaster model. When removed, permits for three-dimensional displacement of respective plaster model as neccessary for model surgery.	Essential
	Rentention disk	Permit for magnetic attachment between plaster models and blue or red plates.	Essential
	paper strips	Plain white paper strips are used to mark the model's sagittal position in relation to blue mounting plate. This is achieved by drawing vertical lines from plaster model to paper strip.The lines are positioned near following regions on each model:• midline• cuspids• first molars• retrotuberal areas	Essential
	Drill and Anchor Bolts	Needed for mounting adjustment rods.	Essential when using rods
	Adjustment rods	May be used to establish an adjustable connection between articulator and plaster models in preparation for intended three-dimensional movements.Also usable as an option to sticking wax when fitting plaster models in best fitting occlusion.	Essential unless triangular spacers are used
	screw driver	To firmly insert rods into anchor bolts.	Essential when using rods
	spanner	To tighten the nuts on the rods.	
	hexagon socket screw key	To tighten the clamps connecting the adjustment rods.	Essential when using rods
	triangular spacers	Triangular spacers of standardized thicknesses (1, 2, 3 and 5 mm) may be used to perform a three-dimensional displacement of the plaster model model surgery.	Essential unless rods are used
	transparent spacer	In cases where no up or down movement of the maxilla is desired, this spacer with its fixed width of 8 mm assures a constant vertical while permittig movements in the sagittal and transversal plane.	Optional
	incisal pin mounted gauge	Used to measure sagittal displacement. The incisors and/or the model base may be used as reference for measurements.	Optional

### Step-by-Step KD-MMS procedure

#### Prerequisites

○ alginate impressions for plaster cast production

▪Special attention needs to be paid to an exact reproduction of the alveolar process, the teeth and occlusal surfaces.

▪The plaster models are to be produced with an extended base approximating the extents of the blue and red plates. This enables the user to apply horizontal and vertical reference lines (see also 2D and 3D planning).

○ arbitrary face bow

▪The standard SAM 2 arbitrary face bow is used to transfer the patient's individual maxillary position in relation to the Axis Orbital Plane.

○ centric bite registration

▪ A centric bite registration is performed in order to obtain a temporomandibular-joint-determined intermaxillary relation [17]. This is especially important in cases with a deviation between habitual occlusion and centric occlusion.

#### 2D-planning

**○ 2D-planning** may be performed ad libitum based on almost any cephalometric analysis. However, in order to transfer the results of 2D-planning to the articulator mounted models, some standardized reference lines [13] are necessary. These are easily integrated into different cephalometric analyses.

#### 3D-planning

○ preparation of SAM 2P articulator

▪yellow mounting plates are removed

▪blue mounting plates are installed

▪red spacers are fitted on blue mounting plates using their built in magnets – in obviously mono-maxillary cases only one red spacer is needed

▪if no customization is desired: set bennett angle to 15 degrees and condyle inclination to 30°

▪if over-correction is desired, condylar inserts may be used

○ the maxillary model is mounted in the articulator using the face bow

○ incisal pin is adjusted to compensate for the centric bite registrate's thickness – usually 2.5 mm are sufficient and small deviations may be tolerated as they do not have any impact on the result

○ the mandibular model is mounted using the centric bite registrate

○ after removal of the bite registrate, the incisal pin is lowered according to the occlusion

○ paper strips are fitted on the blue mounting plates

**Figure 5:**
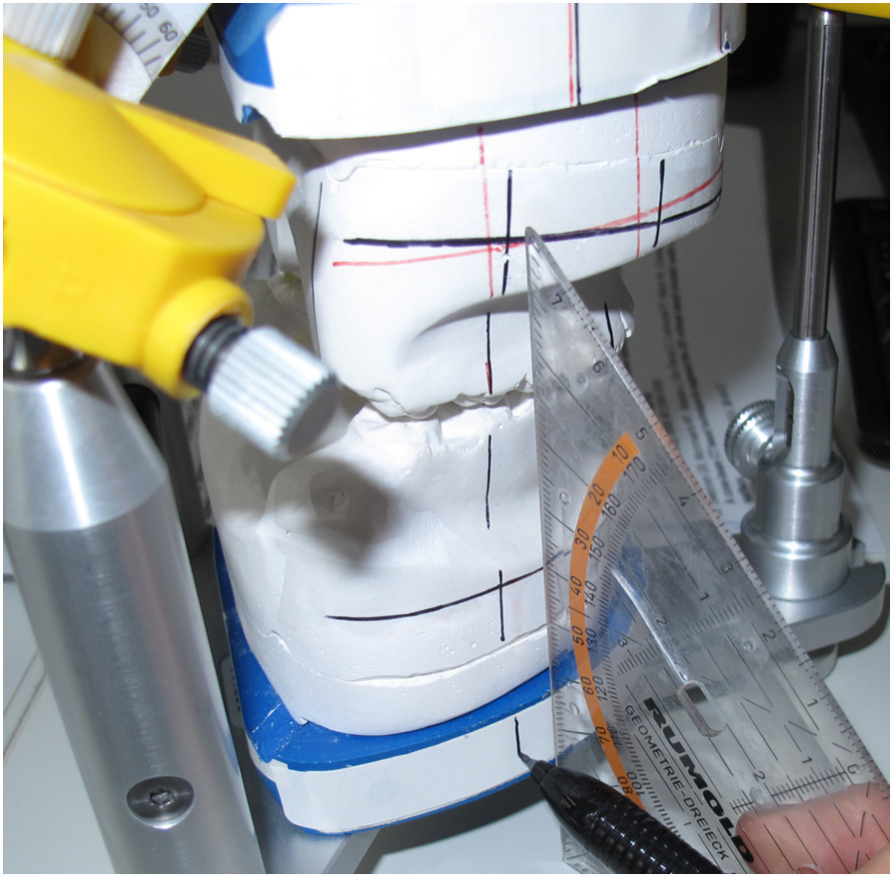
Application of reference lines using a set square.

○ reference lines are drawn vertically from the plaster models to the paper strips using a set square or a similar tool (Figure 5)

○ a horizontal line is drawn on each model in order to approximate the level of osteotomy

▪by default, the line on the maxilla model is applied at half height, which yields an acceptable level of accuracy unless the patient features an extreme short or long faced configuration

**Figure 6:**
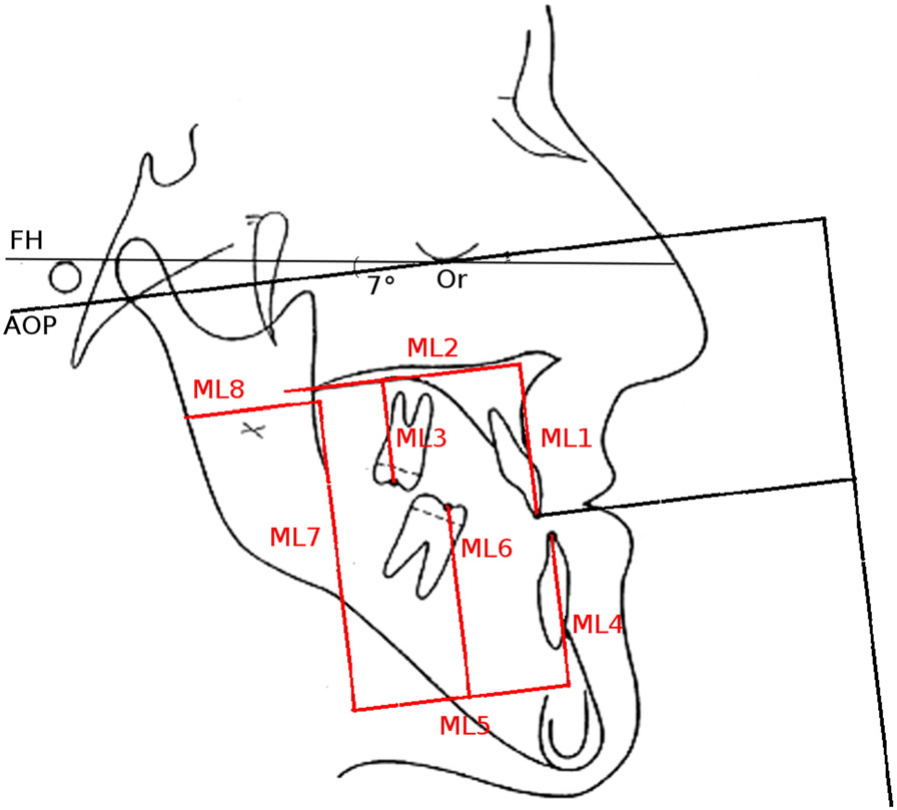
**Axis Orbital Marker Line System (AO-MLS) for approximating maxillary osteotomy locations in anterior (ML1) and posterior (ML3) regions in lateral ceph tracings [**[13]**].**

▪alternatively: individualized placement of horizontal lines in accordance to lateral ceph measurements – reference line ML3 is drawn 5 mm above the first molar root approximating the level of the future osteotomy (Figure 6)

○ the hardware components listed earlier enable the user to conduct model surgery in various ways:

▪model surgery using adjustable rods (instructions for bimaxillary surgery)

•the drill is used to prepare four holes in the maxillary model – to facilliate a stable attachment the holes should be placed approximately level with first molars and cuspids on each side of the model

•anchor bolts are insertedthe mandibular model is attached to the maxillary model in the desired best fitting occlusion either using sticking wax or by the means of additional connecting rods, the latter requiring additional holes to be drilled – using rods istead of wax yields a better overview of the occlusion and leaves the plaster surfaces unstained which is helpful in case of further alternative model surgeries as well as for the subsequent construction of gnathologic splints

**Figure 7:**
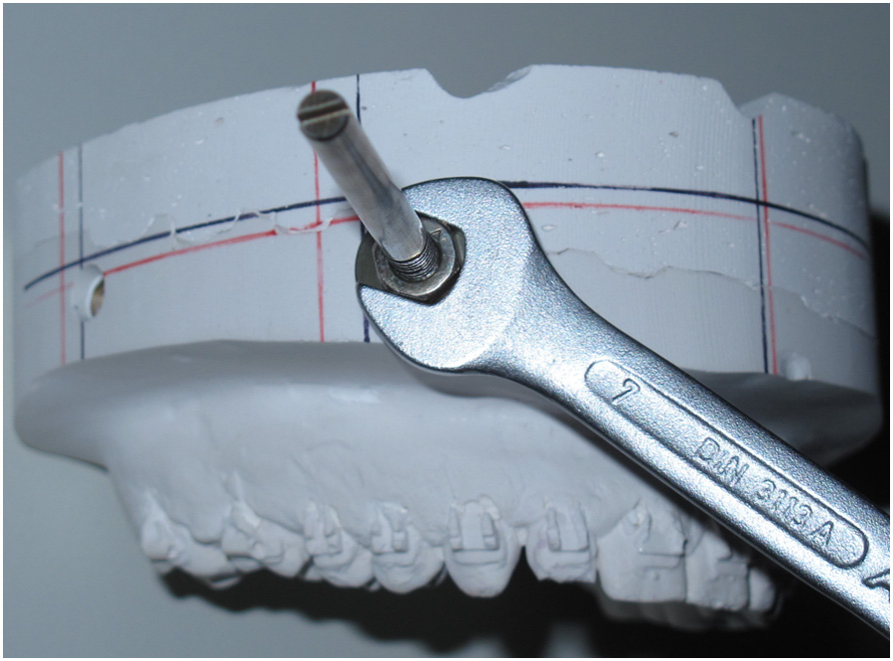
Borings in maxillary plaster cast with adjustment rod inserted into anchor bolt in anterior boring.

•the rods are fixed in the anchor bolts (Figure 7) and in the mandibular blue mounting plate using screw driver and spanner

•the clamps are tightened using the hexagonal socket screw key

•the upper red spacer is removed

•the maxillary model is positioned three-dimensionally according to planned treatment objectives – the previously drawn reference lines permit for a comparison between planned and actual movement, also the incisal pin mounted gauge provides a reference for sagittal positioning

•the base surface of the maxillary model is sprayed with plaster isolation

•the gap between the blue mounting plate and the base of the positioned model is filled with thinly mixed plaster in order to produce a customized socket (maxillary model surgery)

•once the plaster is hardened, the rods connecting the maxillary model with the articulator base (mandibular blue plate) are removed

•the articulator is turned upside down, and another socket is produced in a similar manner to incorporate the displacement of the mandibular complex (mandibular model surgery)

•the rods connecting maxilla and mandibula – or the sticking wax respectively – are removed

•the sockets are separated from the models and trimmed to shape

•models and sockets are left to dry for about 24 hours or placed in a drying chamber

•unless the reference lines drawn in advance of model surgery are still visible, they are applied again (with the red spacers in place)

•additional reference lines are drawn in a different color (red by default) with the custom plaster sockets in place visualizing the displacement

•the displacement is assessed by measuring the distance between black and according red lines permitting for an evaluation of the displacement in the area of the osteotomy sites

•sagittal displacement of the icisors as well as of the maxillary base can be evaluated by using the incisal pin mounted gauge, especially when palatal plane rotations have taken place

▪model surgery using triangular spacer disks (instructions for bimaxillary surgery)

•the positioning of the maxillary model is conducted using the triangular spacer disks. The disks are available in thicknesses of 1, 2, 3 and 5 milimeters enabling the user to combine any thickness between 1 and 8 milimeters with a maximum of two spacer disks

**Figure 8:**
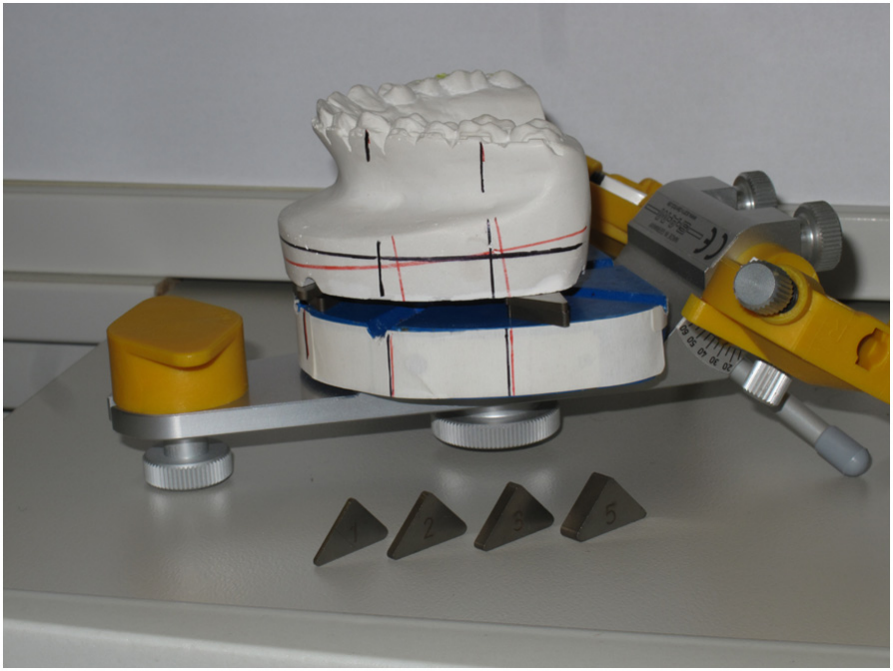
Triangular spacer plates (available in 1, 2, 3 and 5 mm thickness) for positioning of maxillary plaster cast.

•usually three stacks of spacer disks are used: two in the posterior area and one anteriorly determining the necessary vertical displacement (Figure 8)

•the model is moved about on the triangular spacer disks until the planned sagittal and transversal position is established

•the maxillary model with the spacer disks underneath is fixed on the blue mounting plate using sticking wax

•the mandibular model is attached to the maxillary model in the desired best fitting occlusion with sticking wax

•unlike the procedure with rods, the mandibular customized plaster socket is produced first, while the particular procedure of socket production (i. e. insulation, plaster application and trimming) stays the same

•the sticking wax between maxillary model and blue mounting plate is removed without removing the wax connecting maxillary and mandibular model

•then, a maxillary customized plaster socket is fabricated in the usual fashion

•drying, application of reference lines and measurements are conducted as mentioned above

### Model surgery record

**Figure 9:**
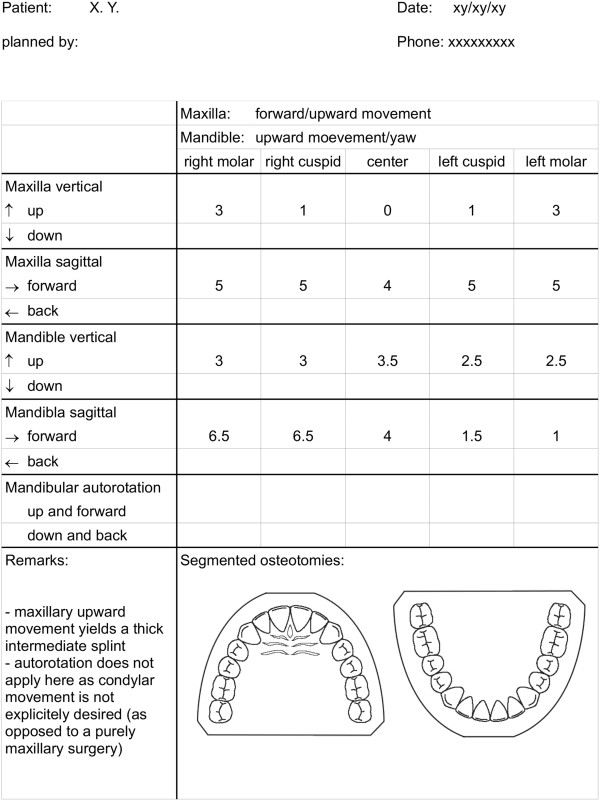
Standardized model surgery record.

The standardized model surgery record provides the surgeon with a summary of the three-dimensional displacements conducted during model surgery. The record form is displayed in Figure 9.

The form permits for an effective discussion of the recorded values and contributes to a precise and standardized overall procedure.

Articulator and model surgery record are routinely present in the operating room and at the surgeon's disposal.

### Gnathologic splint construction

Using the SAM articulator and the according arbitrary face bow registration permits for rotational movements in good axial alignments. This is important as the presence of the splint itself requires the temporomandibular joint to perform a slight opening rotation.

•bimaxillary surgery requires the production of two splints: one intermediate splint and one permanent splint

•if – during actual surgery – the maxilla is to be displaced first, the intermediate splint is fabricated with the red spacer plate underneath the mandibular model and the customized plaster socket on the maxillary model (representing the pre-operative position of the mandible and the post-operative position of the maxilla)

•if the maxilla is to be moved upwards, the resulting space between the dental arches must not be closed by lowering the incisal pin as this would rid the surgeon of his vertical reference, instead, a distinctly thick intermediate splint is to be produced

•the intermediate splint should be made from a different color resin or otherwise marked to make it clearly distinguishable from the permanent splint

•the permanent splint is produced with both customized plaster sockets in place with a slightly raised incisal pin to permit for a minimal yet – in terms of stability - sufficient thickness

•both splints should be tested and cleared of any interferences in opening and closing movements in the articulator

About a day in advance of the surgical procedure, the splints should be checked clinically, i. e. they need to be fitted separately in the patient's maxillary and mandibular dental arch in order to avoid any occlusal disturbances.

In conjunction with the adjustable bone fixation system for sagittal split ramus osteotomy described by Joos [18], an accurate adaptation of the skeletal structures to their splint-determined positions becomes possible.

In addition to its function during surgery, the final splint has also advantageous effects in the post-surgical phase. Firstly, it guides the mandible into its pre-determined position every time the jaws are closed. This is especially important when a semi-rigid bone fixation system as this permits for small, post-surgical corrections through elastic traction of the mandible into the grooves of the splint. Secondly, it creates a most uniform distribution of occlusal contacts. Thereby, it creates favorable conditions for the progress of reossification and reduces the stress on the temporomandibular joints.

## Discussion

Lateral cephalometry can be regarded one of the most frequently used diagnostic procedures for estimating the need for orthognathic surgery as well as the amount of displacement. While other model surgery concepts do take into account the findings obtained from cephalometric measurements, they lack a standardized regime for accurately transferring them onto the articulator. KD-MMS permits for a three-dimensional adaptation of cephalometric measurements by means of the axis orbital marker line system (AO-MLS).

Another advancement could be achieved regarding the need to cut the models used in the planning procedure. The KD-MMS spacer plates enable the user to restore the initial situation or perform various plannings with the same plaster casts. While the system’s development was based on the SAM 2 P, it can be mounted on other articulators and integrated in other model surgery procedures.

Possible future developments are likely to rely on cone beam CT and 3D medical image computing [3,5,19]. However, *“to enable the clinician to make this major paradigm shift in routine planning of orthognathic surgery, both image acquisition systems and 3D virtual planning software must become user-friendly, easily accessible and available at a relatively low cost”*[4].

Some of the error sources [20] inherent in conventional procedures may be eliminated. However, while such approaches are already being pursued, the requirement to impose the planned skeletal displacements onto the operational situs remains. Currently, gnathologic splints and carefully recorded displacement values still need to be excelled by superior methods. Various surgical marking methods, the experience of the surgeon, intraoperative centric guidance and centric fixation are factors which influence overall accuracy [8,9,21,22].

## Conclusions

The KD-MMS hardware components in conjunction with the defined procedures are capable of increasing efficiency and accuracy of model surgery and splint construction. In cases where different surgical approaches need to be evaluated in the course of model surgery, a significant reduction of chair and an optimization of interdisciplinary work flow time may be achieved.

## Competing interests

The authors declare that they have no competing interests.

## Authors’ contributions

UE developed the KDMMS and suggested the original idea for the paper. UE, SF and TZ wrote the main part of the manuscript. UJ and DW reviewed the paper for content, and reviewed and contributed to the writing of all iterations of the paper, including the final version of the manuscript. All authors read and approved the final manuscript.
